# The Functional Efficiency of Older Patients after Lumbar Spine Surgery, the Impact of Pain, and the Passage of Time: Tips for Care Providers

**DOI:** 10.3390/healthcare12171684

**Published:** 2024-08-23

**Authors:** Luiza Mendyka, Sławomir Tobis, Patrycja Talarska-Kulczyk, Dorota Ryszewska-Łabędzka, Magdalena Strugała, Marlena Szewczyczak, Dorota Talarska

**Affiliations:** 1 Department of Preventive Medicine, Faculty of Health Sciences, University of Medical Sciences, 61-701 Poznań, Poland; luizam76@wp.pl (L.M.); magdastrugal@ump.edu.pl (M.S.); mszewczyczak@ump.edu.pl (M.S.); 2 Occupational Therapy Unit, Faculty of Health Sciences, University of Medical Sciences, 61-701 Poznań, Poland; 3 Department of Immunobiology, University of Medical Sciences, 61-701 Poznań, Poland; patrycjatalarska@ump.edu.pl; 4 Department of Nursing, Stanisław Staszic State University of Applied Sciences, 64-920 Piła, Poland; d.labedzka69@gmail.com

**Keywords:** back pain, functioning, elderly people, neurosurgery

## Abstract

Chronic back pain in adults is often a cause of absenteeism from work, limitations in social interactions, and difficulties in performing daily activities. This study aimed to check whether the functioning of everyday activities in elderly patients after neurosurgery improved over time compared to their condition before the procedure and whether there is a connection between fitness, self-esteem, and pain intensity. This was a cross-sectional study carried out using an anonymous survey. This study included patients over 60 years of age from the neurosurgical ward. The following scales were used to assess cognitive functioning, depression, functional status: basic and instrumental activities of daily living (IADL), back pain disability (QBPDS), pain intensity, and self-esteem. The level of independence in terms of IADL and QBPDS increased, and the intensity of pain decreased. Surgery reduced pain intensity from 8 ± 0.8 to 6.1 ± 1.4 points. The level of self-esteem (RSES) remained at a low level. The passage of time after neurosurgical treatment has a beneficial effect on reducing pain intensity and increasing independence in life activities. Daily physical activity and rehabilitation are important factors supporting the return to independence.

## 1. Background

The aging process favors the occurrence of diseases and degenerative changes in the lumbosacral spine [1,2,3]. Degenerative changes contribute to the occurrence of spine pain. The percentage of older people (60 years or older) in almost all developed and developing countries is growing faster than any other age group. Therefore, the number of people with pain in the lumbar spine is significantly increasing [4]. Various factors cause low back pain (LBP). The main causes are lumbosacral discopathy, degenerative changes, osteoporosis, and post-traumatic changes. It also arises and is intensified by factors unrelated to pathological changes, such as excessive emotional tension, depression, hypochondria, and a sedentary lifestyle [5,6]. In Europe, chronic pain most often affects the back (24%), especially the lower part (18%) [7]. It is the leading cause of disability worldwide. In 2020, approximately 1 in 13 people had LBP, which equates to approximately 10% of the world’s population and an increase of 60% compared to 1990 [8]. In a study involving a French population, chronic back pain occurred in 38.3% of the study participants; 59% were women, 15% lost their jobs due to pain, and 67% reported no improvement after pharmacological treatment [7]. In Poland, as many as 44% of complaints related to spinal pain syndrome are located in the lumbosacral section of the spine. Pain that persists for a long time affects the deterioration of general fitness, limits the performance of self-care activities, and reduces the quality of life in various areas, impacting physical and mental well-being, social relationships, and functional capacity [2,3,7,9,10,11,12,13]. Thus, it is a cause of increased social costs incurred by medical and social care [8,9,13].

Spinal stenosis is the most common cause of bothersome LBP symptoms [1,6,11,14,15]. In addition to pain in the buttocks and lower limbs, patients also report symptoms such as intermittent claudication, difficulty moving, numbness, and weakness in the limbs. This clinical syndrome is known as neurogenic claudication [2]. It is accompanied by changes in mental and social functioning, e.g., depression, anxiety, insomnia, worse sexual performance, social isolation, and greater absenteeism from professional activity [6,9,11,12]. In the group of people over 65 years of age with spinal stenosis, migraines, urinary incontinence, hypertension, and arthritis are more common [11]. In cases of severe pain and accompanying neurological symptoms, a decision is made regarding surgical treatment [1,3,16]. Most elderly patients experience positive effects of undergoing surgery, which mainly consists of eliminating or reducing pain and improving everyday functioning [3,11,12].

However, when discharged from the hospital after surgery, they often return to their home environment, dependent on others. It is difficult for elderly people to regain the ability to walk during a short period of hospitalization after surgery. Therefore, due to severe motor dysfunctions, they require support and supervision from their family. However, the positive effect of surgery on an elderly patient depends not only on informal support (from family and friends) but also on formal (institutional) support. Systemic support includes medical and psychological assistance and rehabilitation [8]. Medical education, focused on preparing for self-care, is also an important element of support. It should contain information enabling older people and their loved ones to properly meet their health and hygiene needs in the first period after surgery and further rehabilitation. In the elderly, the goal of rehabilitation is to restore, to the greatest extent possible, the ability to perform basic (ADL) and complex (IADL) activities of daily living [10]. An important element of formal support for patients after the neurosurgical treatment of the L-S spine is sanatorium treatment, which, thanks to systematic rehabilitation, significantly increases independence and improves the quality of life.

Most of the studies published so far concern the epidemiology or quality of life of people with LBP and the complications and functional performance of people after surgery. To a lesser extent, they concern the analysis of the effectiveness of this form of treatment in the elderly. The most frequently expected effect of neurosurgical treatment is increased independence and the disappearance of ailments, primarily pain. Changes occurring in the aging process often reduce the efficiency and effectiveness of the body. Therefore, the effectiveness of surgical treatment in older people in terms of functional efficiency remains a troubling question.

This study aimed to check whether the functioning of everyday activities in elderly patients after neurosurgery improves over time compared to the condition before the procedure and whether there is a connection between fitness, self-esteem, and pain intensity.

## 2. Methods

### 2.1. Participants 

Complete data were obtained from 131 patients in the neurosurgical ward aged 60–75 (Table 1). All patients were hospitalized due to planned lumbar spine surgery.

For all of them, it was their first spinal procedure.

### 2.2. Clinical Situation

In more than half of the group (52.7%), the reason for hospitalization was L4/L5 spinal canal stenosis. Differences in this respect between women and men were statistically insignificant (*p* > 0.05). Another reason was stenosis in the L3/L4 segment (25.2%) and acute sciatica with hernia in the L4/L5 segment (22.1%). Stenosis was more common in women (31.9%) than men (17.7%), and acute sciatica was more common in men (29.0%) than women (15.9%).

Before admission to the hospital, each of the subjects felt pain in their lumbar region. They were taking painkillers due to their ailments. Ketoprofen 3 × 50 mg intravenously was the most frequently (88.5%) taken drug by both men and women after admission to the hospital. Both women (20.3%) and men (21.0%) rarely received Paracetamol (3 × 500 mg dose). Additional drugs taken were Morphine (N = 44, 33.6%), Metamizole (N = 50; 38.2%), and Tramadol (N = 52, 39.7%).

### 2.3. Organization of Research

This was a cross-sectional study. The research was conducted between January 2021 and January 2022.

We offered participation in the study to all consecutively admitted patients in the ward who were qualified for lumbar spine surgery.

This study included patients who agreed to participate in the study and met the inclusion criteria: age 60–75; neurosurgery—degenerative changes, stenosis; no other diseases that impede movement; cognitive performance > 7 AMTS (Abbreviated Mental Test Score) points.

The study group was met 3 times: stage I—one day after the patient was admitted to the ward; stage II—the day the patient was discharged home; stage III—6 weeks after the procedure, during follow-up examinations (Table 2).

Patients between stages II and III of the study participated in a 4-week rehabilitation stay, carried out on a full-time or part-time basis. They underwent inpatient rehabilitation in the musculoskeletal rehabilitation department at the hospital. They came daily for exercises and physiotherapy treatments from home as part of the part-time training.

When qualifying for the study, patients received detailed information on the nature and schedule of the study and the patient’s rights and obligations. Particular attention was paid to the possibility of asking questions and raising issues that were important or disturbing for the patient. All eligible patients were familiarized with “Patient Information” and gave their “Informed Consent” to participate in the study.

This study was approved by the Bioethics Committee at the Medical University of Karol Marcinkowski in Poznań, Poland. The Commission also confirmed that the study did not bear the characteristics of a medical experiment (date 19 December 2018; nr KB 537/24). The research was carried out in accordance with the principles of the Declaration of Helsinki: Ethical Principles of Conducting Medical Research with the Participation of Humans of the World Medical Association.

A thorough analysis of medical documentation of research was preceded. The data were collected using the survey technique. In case of doubts about the questions, the researcher explained their meaning. After the third stage of the study, 131 fully completed questionnaires were obtained, and 15 questionnaires were rejected due to patients’ resignation during the second or third stage of the study. The incomplete completion of the questionnaires was considered as withdrawal from the study.

### 2.4. Measures

1. Anonymous surveys were used to collect demographic and social data.
2. Abbreviated Mental Test Score (AMTS) was used to evaluate cognitive abilities. The scoring range in this scale was from 0 to 10 points. A score below 7 points indicates the presence of cognitive disorders, and 7–10 points indicate normal cognitive functioning [17].
3. Geriatric Depression Scale (GDS) used for comprehensive geriatric assessment. It helps diagnose depression in elderly people. The scoring range is 0–15. A cut-off point was proposed at which the result ≥ 5 indicates depression, and the following ranges indicates severity: 6–9 points for mild and 10–15 for moderate-to-severe depression [18].
4. Barthel Index is a scale used for assessing independence in undertaking basic life activities. Interpretation: 80–100 points—independent, 60–79 points—minimally dependent, 40–59 points partially dependent, 20–39 points very dependent, <20 totally dependent [19].
5. Lawton Instrumental Activities of Daily Living Scale (IADL). We used a version with 9 questions. There are 3 possible answers: 1 point means full dependence, 2 points mean partial dependence, and 3 points mean independence. Point range: 1–27 pts. The higher the points, the greater the independence.
6. The Quebec Back Pain Disability Scale (QBPDS) is a tool designed to assess the degree of functional disability in people with back pain. Each of the 20 activities is scored on a scale of 0 to 5, where 0 = not difficult at all, and 5 = impossible to perform. The range is 20–100 points; and the higher the points, the less fit the person [20].
7. Numerical Rating Scale (NRS). Range: 0 to 10, where 0 is no pain, and 10 is the worst pain imaginable.
8. M. Rosenberg’s Self-Esteem Scale (RSES)—This scale is frequently used and recommended for assessing global self-esteem. It consists of 10 statements to which the respondent reacts by choosing one of four possible answers, according to the Likert scale ranging from 1—strongly agree—to 4—strongly disagree. The total score range for the scale is from 10 to 40 points. During analysis, the responses to positive questions need to be reversed. The general rule for evaluation is that the higher the final score, the better the self-esteem. Given the various methodological approaches to the final results (proportional assessment, distinguishing intervals with different cut-off points), the study adopted the division proposed by earlier researchers: 10–27 low; 28–31 average; >32 high [21].

### 2.5. Statistical Analysis

Depending on the type of variables, various description parameters were used. Measurable variables were described using such parameters as arithmetic mean, standard deviation (SD), median, minimum and maximum values (min. and max.), and qualitative (categorical)—using the categories of number (N) and frequency (%).

The following nonparametric tests were used to test measurable variables:

- Kruskal–Wallis test (H)—to check the significance of differences in three groups (independent samples). To precisely check the significance of the difference between pairs of groups, the Kruskal–Wallis multiple comparison test was used—a post hoc test.
- Mann–Whitney U test (U)—to check the significance of differences in two groups (independent samples).
- Friedman’s ANOVA test (X^2^)—to check the significance of differences in three stages (dependent samples); Dunn’s test (post hoc test) was used to more accurately check the significance of the difference between pairs of steps;
- Wilcoxon’s order of pairs test (Z)—to check the significance of differences in two stages (dependent samples);
- Spearman’s rank correlation coefficient significance test (rs)—to determine correlations between measurable variables;
- A *p*-value < 0.05 was considered statistically significant for all tests. The statistical package STATISTICA 10 PL was used for all calculations.

## 3. Results

The AMTS scale was included in the research criteria, enabling participation in the study. Therefore, it was used only in the first stage of this study. People who scored at least 7 points were qualified for further participation. The average score in the study group is 8.5 ± 1.1 points. The lowest score was 7 points, and the highest was 10.

### 3.1. Assessment of Mental Functioning

On the day of admission, a screening test for depression was performed using the GDS. The average score for the entire group was 5.6 ± 2.3 points (W 5.7; M 5.5 ± 2.3). Mild depression was diagnosed in 51.1% of respondents. This was slightly more common in women (*p* < 0.581) and unemployed people (*p* < 0.001). In the third stage, the average score for the entire study group was 5.2.

### 3.2. Self-Care Scope

The functional status was analyzed using three scales: Barthel Index, IADL, and Quebec scale. Before the procedure, the Barthel Index was 75 ± 8.5 points. On the day of discharge (second stage of the study), participants scored an average of 70 ± 13.5 points, i.e., they were people requiring little help. The range of scores ranged from 45 to 95 points (93.9%).

The IADL scale was used at all stages of this study. The extent of independence significantly increased after surgery compared to the state before surgery (*p* < 0.001). Patients obtained the following: stage I—16.4 ± 1.4 points, stage II—17.4 ± 1.6 points, stage III—25.2 ± 2.0 points. Before surgery, the patients had the most difficulties with activities requiring mobility, especially with shopping or going up and down the stairs.

In the first, second, and third stages of the research, the QBPDS scale was used (Table 3). This scale enables the assessment of the functioning of a patient with spinal complaints in terms of basic and complex everyday activities, which is why it was used to compare the results with the IADL and the Barthel Index. With the passage of time, as in IADL, an increase in the independence of the respondents was demonstrated. Before the procedure, the average score was 62.1 ± 7.1 points. In the third stage of the study, it was 44.9 ± 8.1 points.

Before the procedure, the study group had difficulties primarily in performing activities related to moving over longer distances (over a kilometer), reaching objects from higher shelves, and bending and carrying heavier things. Six weeks after the procedure, the respondents’ efficiency increased, especially in climbing stairs, walking a distance of 400 m, and performing activities resulting from everyday activity, such as putting on socks, taking food from the refrigerator, or cleaning in a bent position.

### 3.3. The Level of Self-Esteem and Pain Intensity

The level of self-esteem (RSES) changed minimally over time (before the procedure—26.7 ± 3.0; 6 weeks after the procedure 26.9 ± 2.9) but was statistically significant (Z 3.64; *p* < 0.001). Pain intensity (NRS) was measured at all stages of the study. Pain significantly decreased after surgery (X^2^ 158.9, *p* < 0.001). Before surgery, the average intensity level in the study group was 8.0 ± 0.8 points; on the day of discharge, 7.3 ± 0.9 points; on the third stage, 6.1 ± 1.4 points. There was no significant difference in pain intensity after considering gender and age (*p* > 0.05).

To identify factors influencing the functioning of people after spinal surgery, the analysis took into account demographic and social factors, such as the passage of time, GDS, pain intensity, and self-esteem. Socio-demographic factors did not significantly affect the functional status of the study group. An association was only found between the RSES results and the type of living (H 8.17; *p* = 0.017). Higher self-esteem was found in people living alone (27.3 ± 2.3 points) than with a spouse/partner (26.9 ± 3.1 points), and between GDS and professional activity (U −2.04; *p* < 0.041). Fewer symptoms of depression occurred in people who were working. The analysis confirmed the correlation between self-esteem (RSES) and the severity of depression symptoms (GDS, Rs −0.593; *p* < 0.001). Higher self-esteem occurred in people with less severe symptoms of depression.

**Figure 1:**
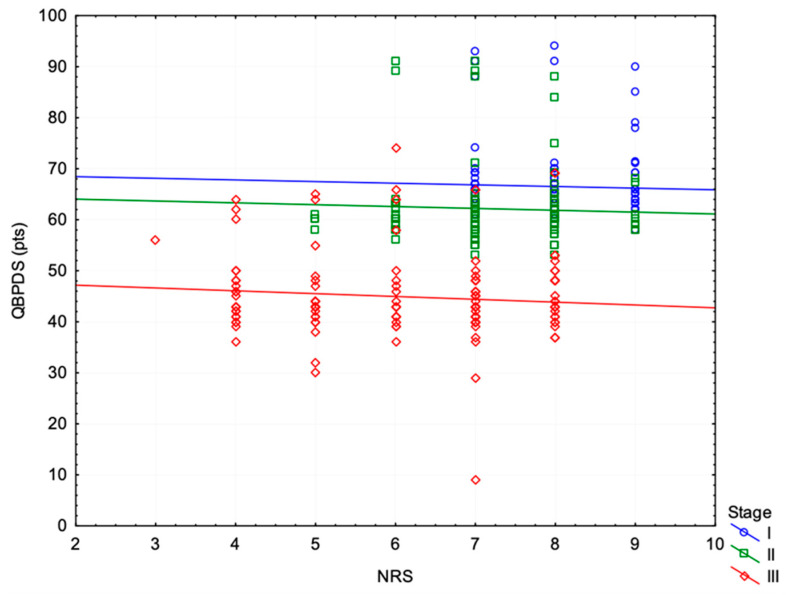
Cumulative scatterplot between NRS and QBPDS in stages I, II, and III.

A multiple regression analysis was performed to confirm the relationship between independence in undertaking life activities (QBPDS) and the examination study and the intensity of pain at individual stages of examination. The multiple regression model for the variable functional status (QBPDS score), taking into account the independent variable pain intensity and stage of testing, turned out to be statistically significant (*p* < 0.001). For the dependent variable of QBPDS, the variable research stage was a significant coefficient (b* −0.76 ± 0.04; *p* < 0.001). The regression model explained 55.8% of the variation in QBPDS. Increasing the stage by one level reduces the points on the QBPDS scale by an average of 10.97 ± 0.60 points, with the remaining variable remaining constant (Figure 1).

The beneficial effect of increasing independence was confirmed by obtaining a correlation (*p* < 0.006) between the RSES result and the level of motor skills (QBPDS result) at the third stage of the study. Higher self-esteem on the RSES was accompanied by greater patient performance on the QBPDS.

## 4. Discussion

The good effects obtained after neurosurgical treatment in elderly people also contribute to an increase in surgical procedures in the oldest age group [11]. The benefits mentioned by both patients and doctors include reducing pain, increasing the range of mobility, and improving the quality of life [12,13,22].

Lumbar spine stenosis causes various troublesome ailments that significantly limit the daily functioning of older people and negatively affect their quality of life [3,4,6,11,12]. They impair the quality of life more than diabetes, heart disease, arthritis, or stroke [11]. Lower back pain is often caused by climbing stairs, bending and kneeling, moving long distances, and performing housework and shopping [9]. Good effects in eliminating pain in the lumbar spine are achieved by limiting sitting time, extending the time spent on high- and moderate-intensity activities, and systematically undertaking stretching, strengthening, and muscle conditioning exercises aimed at improving the overall fitness of the back and lower limbs as well as facilitating the flexion of the lumbar spine [2,5]. Edwards et al. [10] showed that for older people with lumbar spinal stenosis, apart from pain and difficulty in movement, an important aspect was the inability to exercise and limited participation in recreational activities. These two forms of activity probably provided social contact for older people, which was greatly limited due to pain. If there are no satisfactory results after pharmacological treatment and rehabilitation, a decision is made about neurosurgical treatment.

Spine surgery in the elderly, especially in the older age group, is associated with an increased risk of perioperative complications. Still, modern surgical techniques and good patient preparation (compensation for deficiencies and diseases) increase procedure safety [23]. In a Korean study, only 15% of patients experienced complications after spinal fusion surgery, more often in the older age group [1]. The potential complications mentioned include increased pain, nerve damage, infections, need for reoperation, and death [13].

Reducing the Oswestry disability index in a group of Canadian patients with hypertension from 58.9 points before the procedure to 30.1 after the procedure and from 59.0 to 28.7 points in the group without hypertension confirms the sound effects of neurosurgical treatment [15]. Positive effects are also noticed in patients with severe pain [1,15,23]. In the study by Rampersaud et al. [24], 49.8% of respondents stated that their pre-surgery expectations had been met, while 32.9% believed that their most important expectation had not been met. Respondents primarily expected the disappearance or reduction in pain in arms, legs, and back, as well as improved general fitness, independence in basic life activities, increased participation in sports and recreational activities, and improved mental state [24]. People with higher expectations rarely reported that their expectations were met. However, reducing disability and pain often contributed to meeting expectations [24].

A common persistent symptom after the procedure is leg pain and sensory disturbances [14]. These ailments cause kinesiophobia which increases disability and thus worsens the quality of life of elderly people [14,25]. Greater effects after surgical treatment were observed in people with better health status before the procedure, who were more functionally fit, and those referred to post-hospital rehabilitation [13].

Earlier return to functional fitness allows for a quick introduction of strength training after surgery and its systematic implementation [26]. Combining cognitive therapy with exercises also has a beneficial effect [25]. However, the most positive effect is seen in people undertaking daily activities resulting from household chores and performing exercises at home [22,27]. In our study, we also found that the independence of the study group improved over time. The increase in fitness resulted from both physiotherapeutic treatments and exercises performed during the rehabilitation period between the second and third stages of the study, as well as systematic activities undertaken at home, such as tidying up, cooking, shopping, etc. Research conducted by Iranian scientists in three periods, before surgery and 6 and 12 months after surgery, showed significant improvement in patients’ functioning over time after surgery [3]. In the study group, leg and back pain decreased, and the quality of life increased. A higher improvement in the quality of life after the procedure was found in the group aged 61–70, while a reduction in pain was observed in people aged 30–40 [3]. Other study authors found better functional effects in patients months after surgery [3,12,16]. In one study, three months after the procedure, patients confirmed improvement in their functioning in the physical dimension of HRQOL, although they still reported excessive fatigue and loss of vigor [12].

Our study measured pain intensity (NRS) at all stages. The pain made it difficult to perform activities requiring movement. A correlation was found between pain intensity and independence in undertaking daily activities (QBPDS). However, both before the procedure and 6 months after the procedure, back pain was not related to socio-demographic factors. Different results were obtained in a French study, which showed that chronic back pain was inversely related to education. This was more frequently experienced by people with lower education and farmers [7]. Occupational history also played a role in chronic back pain. Blue-collar workers and unemployed individuals experienced chronic pain more often than white-collar workers [7].

Depression and anxiety are highly comorbid with chronic pain and can significantly impact the experience of pain, leading to greater pain intensity, impaired functioning, and reduced quality of life [28]. A large percentage of patients remain depressed after surgery on the lower spine. In our research, half of the study group had moderate symptoms of depression before surgery. After the procedure, depressed mood occurred in 1/3 (31.3%) of the group. This result confirms the need for holistic support for patients after surgery.

Chronic pain is also associated with low self-esteem [29]. Our research has shown a connection between the level of independence (QBPDS scale) and self-perception (RSES). Higher self-esteem on the RSES was accompanied by greater patient performance on the QBPDS.

Research conducted by Sousa et al. [4] showed that people with a more resistant phenotype (better adaptation syndrome) may have a greater sense of coherence, which allows them to mobilize resources to cope with current pain-related challenges. Pain among older people occurs as part of aging and becomes a natural part of life. Therefore, it becomes less upsetting or is ignored [4]. Older people are more likely to mobilize to perform everyday activities despite the pain they feel. Therefore, in the therapeutic treatment, it should be remembered that there is a high level of subjectivity in assessing the results of spinal surgery performed by older people [30].

Our study showed that an important element of the analysis of the effects of surgery is the assessment of the functional status before surgery, during which attention should be paid to the intensity of symptoms and functional limitations. Individually selected methods of comprehensive therapy, e.g., surgery, rehabilitation, psychotherapy, and medical education, provide a chance to increase independence and reduce troublesome symptoms. An important element in the process of improving functional ability is rehabilitation. To be effective, it should be tailored individually and adjusted to the patient’s needs. The effectiveness of the therapy should be monitored during the rehabilitation process, and changes should be made if necessary [31].

An important aspect is informing the patient before surgery about functional limitations that may occur after the procedure and the need to obtain care support from family or friends. The patient and loved ones should also know that regular exercise and daily activities enable faster recovery and better self-perception.

A certain limitation was the small group of patients, one location for conducting research and the relatively short observation period, which was six weeks post-procedure. In subsequent studies, with a larger group of patients, an analysis of the effectiveness of the procedure, i.e., the resolution of ailments, should be undertaken, taking into account the age of the patients and the severity of the lesions before the procedure and the scope of rehabilitation activities after the procedure. Other factors influencing the effects of the procedure should also be considered, e.g., body weight, comorbidities, and duration of ailments, before the procedure.

The obtained results show that pain adversely affects not only the level of independence but also the mental state of patients. Therefore, it is important to consider the mood assessment in patients with chronic back pain.

## 5. Conclusions

The passage of time after neurosurgical treatment has a beneficial effect on reducing pain intensity and increasing independence in life activities. Daily physical activity and rehabilitation are important factors supporting the return to independence. Increased independence helps improve self-perception.

## Figures and Tables

**Table 1:** Socio-demographic characteristics of the study group.

Variable	Women, N (%)	Men, N (%)	Total, N (%)
Sex	69 (52.7)	62 (47.3)	131 (100)
Age—mean	69.9 ± 2.7	70.2 ± 30	70.0 ± 2.8
Education—number of years of education	11.4 ± 2.6	11.3 ± 3.1	11.4 ± 2.8
Marital status:			
- single	11 (15.9)	8 (12.9)	19 (14.5)
- with a partner	58 (84.1)	54 (87.1)	112 (85.5)
Financial situation:			
- good	51 (73.9)	44 (71.0)	95 (72.5)
- bad	18 (26.1)	18 (29.0)	36 (27.5)
Professional activity:			
- retirement pension	34 (49.3)	36 (58.1)	70 (53.4)
- disability pension	2 (2.9)	0 (0.0)	2 (1.5)
- employed	33 (47.8)	26 (41.9)	59 (45.0)
Having a guardian:			
- yes	0 (0.0)	0 (0.0)	0 (0.0)
- no	69 (100.0)	62 (100.0)	131 (100.0)

**Table 2:** List of scales used in particular stages of research.

Stage	Place of Examination	Deadline for Conducting Research Activities	Research Tools *
I	Neurosurgery department	The first day after admission to the ward	AMTS, GDS, Barthel Index, IADL, QBPDS, RSES, NRS.
II	Neurosurgery department	Day of discharge	Barthel Index, IADL, NRS, QBPDS.
III	Neurosurgery outpatient clinic	Six weeks after surgery, during follow-up examinations	IADL, GDS, RSES, NRS, QBPDS.

* Abbreviated Mental Test Score (AMTS), Geriatric Depression Scale (GDS), Barthel Index, Lawton Instrumental Activities of Daily Living Scale (IADL), Quebec Back Pain Disability Scale (QBPDS), Numerical Rating Scale (NRS), M. Rosenberg’s Self-Esteem Scale (RSES).

**Table 3:** Assessment of patients’ independence before and after surgery—the QBPDS.

Quebec Back Pain Disability Scale	I N = 131		III N = 131		Z	*p* *
	Mean	SD	Mean	SD		
1. Getting out of bed	2.67	0.49	2.13	0.76	5.96	<0.0001
2. Sleeping all night	0.89	0.56	0.71	0.61	3.61	0.0003
3. Turning over in bed	2.67	0.67	1.37	0.76	8.98	<0.0001
4. Driving a car	1.50	1.79	1.40	1.74	2.10	0.0357
5. Standing for 20–30 min	4.17	0.53	3.24	0.64	8.72	<0.0001
6. Sitting on a chair for a few hours	3.78	0.84	3.37	0.81	5.09	<0.0001
7. Going up one flight of stairs	4.37	0.65	1.80	1.06	9.74	<0.0001
8. Walking about 300–400 m	3.55	0.79	1.25	1.12	9.39	<0.0001
9. Walking a few kilometers	5.00	0.00	3.75	0.62	9.62	<0.0001
10. Reaching higher shelves	5.00	0.00	4.02	0.40	9.59	<0.0001
11. Throwing a ball	5.00	0.00	3.24	0.81	9.62	<0.0001
12. Running about 100 m	5.00	0.00	4.23	0.46	8.64	<0.0001
13. Taking food out of the refrigerator	0.26	1.07	0.09	0.64	2.02	0.0431
14. Making the bed	0.43	1.17	0.17	0.45	3.18	0.0015
15. Putting on socks	2.42	0.89	1.11	0.75	9.02	<0.0001
16. Leaning over to wash the tub	4.98	0.26	4.02	0.49	9.55	<0.0001
17. Moving a chair	0.27	1.00	0.11	0.67	2.52	0.0117
18. Pulling or pushing heavy doors	1.57	1.20	1.38	0.99	3.30	0.0010
19. Carrying two shopping bags	3.57	0.96	3.19	0.88	4.66	<0.0001
20. Picking up and carrying a heavy suitcase	5.00	0.00	4.31	1.83	8.34	<0.0001

* the result of the Wilcoxon order of pairs test.

## Data Availability

The original contributions presented in this study are included in the article, and further inquiries can be directed to the corresponding author.
